# Patient satisfaction and ethics in a public hospital practice

**DOI:** 10.4103/0970-0358.44919

**Published:** 2008

**Authors:** Mukund Jagannathan

**Affiliations:** Head Department of Plastic Surgery, Lokmanya Tilak Municipal General Hospital, Sion, Mumbai-400 022, India. E-mail: mukund.jagannathan@gmail.com

Patient satisfaction is a very vaguely defined, yet definite term used in evaluation of results. Unlike many other branches of medicine, Plastic Surgeons have one additional factor to deal with, and that is the subjective evaluation of results, as perceived by the surgeon, the patient, the relatives, and others. In a society like ours, there are several people involved in decision making in respect to elective surgery. Financial and emotional independence are still a small distance away. Which is probably we don't see too many cases of facelifts. Based on our skin types, our population tends to really need major facial rejuvenation at a later age as compared to western populations, and not too many women (especially) are financially and emotionally independent at this stage in life.

Being in a lucky (?) situation of having a dual practice (public hospital/teaching and limited private practice) I am in a position to evaluate both spheres with a view to analyzing eventual patient satisfaction. I must add here that patient satisfaction is the single most important criterion that needs to be addressed, irrespective of the nature of practice. This is the central pillar around which our unit operates, and this has been conveyed in no uncertain terms to my residents and staff.

Unfortunately, patients attending our general OPD are usually perceived to be there by necessity, and not design. This could be due to a variety of reasons including distance and financial limitations. It is our aim that in addition to these, we should attract patients because of our results, our approach, our empathy, and by word of mouth from other satisfied patients.

Here are a few scenarios, which may help to place things in perspective.

1. A burns victim (lady) had severe scarring on the face. It was decided to expand the deltopectoral areas and transfer the skin as a flap. She was assisted by the social worker for purchasing tissue expanders, appointments were given early by jumping the queue, and she was given more than the normal share of attention (normal by public hospital standards, a term which in itself is not appropriate). She got what we felt was a perfectly acceptable result. She was told that there would be revisions, thinning and the like, and she was fine with that. One day she committed suicide, and that was it. There was no forewarning or sign, it just happened.
2. A colleague in the hospital brought her sister, who did not like her nose. She had a rather difficult nose, and right from the first consultation, we were on the defensive as far as the result was concerned. All we promised was that we would do our best, and while we could expect an improvement, it would not be perfect. She came all the way from North India, and was very keen to get operated the next day. She had already sent her photographs in advance and spoken telephonically. We did the procedure, and she was here for a few days postoperatively. The result was average, but she called up after 3 months, sent her postoperative photos, without us asking for them, and was bubbling with enthusiasm. The surgery had made a tremendous difference to her confidence and her life, and she got married shortly after that. We were really amazed, as the result was not that good in our own assessment.
3. A patient had an extensive low flow malformation involving one side of the face. It was grotesque, and he already had been operated elsewhere a couple of times with no result. We told him that it was too extensive and would not operate. He was insistent, and was even prepared for death on table. He was going into severe depression. We still did not operate, instead we showed him photos of patients with worse deformities, and how they were living with them. We arranged for one such patient to meet with him, and they had a dialogue, and he was a changed man. He accepted his condition, and learned to live with it. We were extremely careful not to use any derogatory terms while discussing his deformity, or secluding him during the consultation. In fact we kept him among several patients, and cheerfully discussed his situation as though it were routine. We never tried to play down the deformity either. We just called it as it was.

In a public hospital, we have realized that patient satisfaction is dependent on several factors in addition to surgical results. It is the way you talk to the patient, the degree of importance that is given to his or her problem, the amount of time you spend with them, the amount that you handle personally rather than delegating it to your juniors, and the way you handle the relatives. Other peculiar factors are also seen from time to time. For example, we have noticed that patients philosophically accept a failure of a tissue expansion process when a new expander is used, but are unhappy with failure when an old expander is reused. This factor also comes into play when a variety of implants of varying costs are available for use. A failure in a top of the line implant is attributed to fate, whereas a lesser implant is blamed if there is a problem.

Mental status and surgical course of a patient are inexorably related. The following example makes it clear.

A 35 year old healthy female had a localized soft tissue tumour in the infraclavicular region, requiring wide excision and a local flap. She was explained the procedure and was also told that there was no alternative treatment available. She was extremely apprehensive and kept asking whether anything could go wrong. We explained that general anaesthesia has its complications, but the incidence in a healthy person was very negligible. However she was still very scared and developed all sorts of ectopic beats, tachyarrhythmia and what not during induction. We postponed the case, waited till she was fully conscious, and literally held her hand, and told her what had happened. We urged her to have a more positive outlook. We told her to go home for a couple of days and come back when she was relaxed. She was operated without a hitch after 3 to 4 days. Surgery was uneventful and everything was perfect.

We have attempted to analyse these beliefs, expectations and outcomes, and have come up with a code of conduct while dealing with large numbers of patients who throng the OPDs.

1. Treat every general OPD patient like a private patient. Though there is no incentive monetarily, there is a greater level of satisfaction when dealing with a friendly patient. Similarly, patients respond better to treatment and are able to handle minor and major negative sequelae better.
2. Remember that the patient is the central figure around which your day revolves. He/she is the reason you are there. Your loyalty to him/her must be unquestioned. Everything else comes second. These statements were made by Dr. A. D. Dias, our erstwhile Head of Department, and they should serve as a guiding principle. Talk as gently as possible. On several occasions, the patient does not understand the large amount of information that has been handed out to him/her. Sometimes it may have to be reinforced in simpler terms. Residents get agitated when the patient does not understand what seems to them, a simple explanation. They try to get rid of the patient saying that they would explain later (in short don't waste the boss's time). This practice is also detrimental to the confidence levels of a patient. On several occasions, we have called the patient a second time, for a further consultation.
3. We run a departmental development fund, all receipted and above board. We encourage patients to make contributions. However the standing instructions are that the topic is to be broached ONLY after the patient has finished the treatment (successfully) and is on the point of going home. It is NEVER to be mentioned at the time of appointment or prior to surgery. The patient will assume that it is the pre-requisite of a successful surgery, and if something goes wrong, will blame it on this fact. Residents feel that once treatment is over, the patient will not feel obliged to donate. So be it. It is infinitely preferable to the other scenario.
4. Sometimes, there is friction between a patient and one staff member. It reaches a stage when both have lost confidence in each other. It happens, and neither party may be completely at fault. In such situations, again, there are standing instructions to refer the patient to another staff member. The point is that the patient has to be brought to a satisfactory mental status prior to definitive treatment.
5. When giving options for treatment, give all the necessary information without demeaning any particular option. This is especially important when dealing with a variety of implants. We advise them about stainless steel, indigenous titanium and imported titanium implants. Since the cost factor is significant, while giving options, we mention advantages of the more expensive implants with respect to ease of use and the time saved. At no time do we say that stainless steel will not work. After all we have used it for more than 15 years. If you hint that steel, or Indian titanium is inferior, they will never accept its use, and there is a constant worry even after the surgery is done with these. The words of a doctor, when a poor patient has come by necessity to a public hospital, are of paramount importance. Therefore, one must use words very carefully.
6. If there is a mishap, either major or minor, do not bully your way through the situation. Always be gentle and sympathetic. Accept your portion of the blame. I don't mean that you have to use words like ‘I screwed up’ or equivalents, but be humble, and make it clear that both you and the patient are together in this situation, and that the best solution should be found. This will go a long way in restorative surgery and will universally find greater acceptance of results.
  Gradinger and Courtiss in their chapter “Analyses of the Aesthetic Surgery Patient”[1] have postulated three types of suboptimal results.
  - Patient happy, surgeon unhappy
  - Patient and surgeon unhappy
  - Surgeon happy, patient unhappy

- In this situation, the significant thing is that the patient is happy. The surgeon's unhappiness can have several reasons, but it is still the best situation to be in.
- In this situation, there is at least a common direction in which to go. Judicious re operation, referral to a senior colleague, repeated reassurances all go a long way in dealing with this problem.
- This is the worst scenario. There is likely to be a breakdown of communications. Probably the most judicious thing to do would be to refer the case, not by getting rid of the patient, but by being involved in the discussion with the next surgeon.

We are willing to accept situation A for every case, as long as B and C are not seen in our practice. Situation A may not make for surgical reporting, or paper publication, or it may even give us a sense of unease, wondering what we did wrong, but believe me, it is infinitely safer.

There are very few absolutes in our practice. Merely by being more responsive and friendly to our patients, and treating them with a good degree of empathy and patience, we can make the degree of acceptance much greater. I realize that in a busy general hospital, time is a luxury that sometimes is unaffordable, but we must make the effort.
